# Cultivation of ammonia-oxidising archaea on solid medium

**DOI:** 10.1093/femsle/fnac029

**Published:** 2022-03-22

**Authors:** Timothy Klein, Lianna Poghosyan, J Elaine Barclay, J Colin Murrell, Matthew I Hutchings, Laura E Lehtovirta-Morley

**Affiliations:** School of Biological Sciences, University of East Anglia, Norwich Research Park, Norwich, NR4 7TJ, UK; School of Biological Sciences, University of East Anglia, Norwich Research Park, Norwich, NR4 7TJ, UK; Bioimaging Facility, John Innes Centre, Norwich Research Park, Norwich, NR4 7UH, UK; School of Environmental Sciences, University of East Anglia, Norwich Research Park, Norwich, NR4 7TJ, UK; Department of Molecular Microbiology, John Innes Centre, Norwich Research Park, Norwich, NR4 7UH, UK; School of Biological Sciences, University of East Anglia, Norwich Research Park, Norwich, NR4 7TJ, UK

**Keywords:** ammonia-oxidising archaea, solid medium, cultivation, colonies, solidifying agent, Phytagel

## Abstract

Ammonia-oxidising archaea (AOA) are environmentally important microorganisms involved in the biogeochemical cycling of nitrogen. Routine cultivation of AOA is exclusively performed in liquid cultures and reports on their growth on solid medium are scarce. The ability to grow AOA on solid medium would be beneficial for not only the purification of enrichment cultures but also for developing genetic tools. The aim of this study was to develop a reliable method for growing individual colonies from AOA cultures on solid medium. Three phylogenetically distinct AOA strains were tested: ‘*Candidatus* Nitrosocosmicus franklandus C13’, *Nitrososphaera viennensis* EN76 and ‘*Candidatus* Nitrosotalea sinensis Nd2’. Of the gelling agents tested, agar and Bacto-agar severely inhibited growth of all three strains. In contrast, both *‘Ca*. N. franklandus C13’ and *N. viennensis* EN76 tolerated Phytagel™ while the acidophilic *‘Ca*. N. sinensis Nd2’ was completely inhibited. Based on these observations, we developed a Liquid-Solid (LS) method that involves immobilising cells in Phytagel™ and overlaying with liquid medium. This approach resulted in the development of visible distinct colonies from *‘Ca*. N. franklandus C13’ and *N. viennensis* EN76 cultures and lays the groundwork for the genetic manipulation of this group of microorganisms.

## Teaser

A novel method for growing ammonia-oxidising archaea as single colonies on solid medium.

## Introduction

Ammonia-oxidising archaea (AOA) comprise an important group of microorganisms affiliated with the phylum Thermoproteota, formerly known as Thaumarchaeota (Brochier-Armanet *et al*.^2008^, Rinke *et al*.^2021^). AOA are involved in the oxidation of ammonia to nitrite, the first and rate-limiting step of nitrification. The role of AOA in nitrification coupled with their ubiquitous distribution in both marine and terrestrial ecosystems, highlights their pivotal role in biogeochemical cycling of nitrogen (de la Torre *et al*. 2008, Wang *et al*. 2017, Alves *et al*. 2018, Beeckman *et al*. 2018). While nitrification is vital for nitrogen cycling, it is also associated with nitrogen fertilizer loss and leaching of nitrites and nitrates into aquatic systems leading to eutrophication (Raun and Johnson 1999). Thus, there is an urgent need to further explore AOA and the molecular mechanisms underpinning their physiology.

Since the discovery and isolation of the first AOA strain *Nitrosopumilus maritimus* SCM1 in 2005, our understanding of AOA has steadily expanded through culture-dependent and culture-independent approaches (Könneke *et al*. 2005). Nonetheless, large gaps in our knowledge of the biochemistry, physiology and niche adaptation of AOA still exist due to limitations to the currently available methods.

Culture-independent methods, which mainly comprise of ‘OMICS’-based approaches, provide valuable insights into the possible functional roles of AOA in the environment. However, the high percentage of archaeal genes of unknown function and the lack of comprehensive nucleic acid and protein databases, limit the ability to decipher molecular pathways in AOA (Marakova *et al*. 2019). This is exemplified by current efforts to elucidate the archaeal ammonia oxidation pathway which, based on genomic and biochemical studies, differs considerably from that of ammonia-oxidising bacteria (Lehtovirta-Morley 2018).

In many microorganisms, gene function is investigated with knock-out/knock-in genetic experiments. However, this approach is currently not feasible for AOA because a genetic system has not yet been developed. This is a major stumbling block in exploring and validating the hypotheses generated from metagenomics, proteomics and transcriptomics data.

A major limiting factor in the development of a genetic system for AOA is the inability to routinely grow them on solid medium as single colonies (Leigh *et al*. 2011, Kohler and Metcalf 2012). Single colonies represent a clonal population of cells and facilitate the precise analysis of specific genotypes. Currently, AOA are grown in liquid cultures and reports on their cultivation on solid medium are limited to a single publication by Chu *et al*. (2015) who demonstrated the growth of *Nitrosopumilus* sp. AR in low-melting point agarose. However, the growth observed in that study was a continuous mass of cells and no distinct colonies were evident. While the reasons for the limited success in growing AOA on solid medium are unclear, the role of solidifying agents on microbial growth is often overlooked (Janssen *et al*. 2002, Tamaki *et al*. 2009).

For instance, Tanaka *et al*. (2014) demonstrated agar to be inhibitory for microbial growth due to the production of hydrogen peroxide when agar was autoclaved together with phosphates. The inhibition of colony formation of the chemolithoautotroph *Thiobacillus ferrooxidans* on agar has also previously been reported and is attributed to the release of free sugars (i.e. D-galactose) from agar at low pH (Tuovinen and Kelly 1973). Microbiologists have also experimented with alternative solidifying agents such as gellan gum, resulting in the isolation of novel and previously uncultured microorganisms of the Phylum Verrucomicrobia (Tamaki *et al*. 2005). It is therefore evident that the choice of solidifying agents is an important consideration for successful cultivation of microbes on solid medium.

The aim of this study was to develop a novel method for the cultivation of AOA on solid medium. We hypothesised that the choice of gelling agent is a key factor to consider when growing AOA on solid medium.

## Materials and methods

### Archaeal strains and cultivation conditions

The AOA strains used in this study, ‘*Ca*. Nitrosocosmicus franklandus C13’, *Nitrososphaera viennensis* EN76 and ‘Ca. Nitrosotalea sinensis Nd2’ were routinely maintained in liquid fresh-water medium (FWM) as previously described (Tourna *et al*. 2011, Lehtovirta-Morley *et al*. 2014, Lehtovirta-Morley *et al*. 2016). The pH indicator, phenol red, was added to the medium of both neutrophilic AOA strains at a final concentration of 1.4 μM. All cultures were incubated at 37°C in the dark under static conditions. Cultures were regularly monitored for the presence of heterotrophic bacteria by plating on LB and R2A agar plates.

### Nitrite measurements

Growth of AOA cultures was monitored by measuring nitrite accumulation. Nitrite concentration in the culture medium was measured using the Greiss colorimetric assay with sulphanilamide and N-(1-naphthyl) ethylenediamide in a 96-well plate format as previously described (Lehtovirta-Morley *et al*. 2016). Absorbance was measured at 540 nm using a VersaMax^™^ plate reader (Molecular Devices).

### Assessing the effect of solidifying agents on the growth of AOA

The inoculum was prepared by harvesting 500 ml of a mid-late exponential liquid culture onto a 0.2 µM membrane filter (PES, Millipore). Cells were washed on the membrane with 50–100 ml of sterile FWM basal salts. The membrane filter was subsequently transferred into 10 ml of FWM basal salts and briefly vortexed. This cell suspension was serially diluted (1:10) as required.

To assess the effects of 1.5% (w/v) Agar (Formedium, UK), 1.5% (w/v) Bacto-Agar (BD Difco™), 1.4% (w/v) Noble-Agar (BD Difco™), 1.4% (w/v) Agarose (Melford, UK) and 1.7% (w/v) Phytagel™ (Sigma-Aldrich®) on the growth of AOA, a biphasic mixture of the gel and liquid medium was prepared. Briefly, to each 30 ml plastic screw-cap vial (Greiner Bio-One), 10 ml of the molten gelling agent (containing all medium supplements) was added and allowed to solidify. The solid layer was subsequently overlayed with an equal volume of liquid medium and inoculated with 80–100 µl of a diluted cell suspension (10^−1^, ∼10^6^ cells mL ^−1^) as described above. All experiments were performed in triplicate and control experiments were performed in the absence of a gelling agent. Vials were incubated in the dark at 37°C under static conditions.

### Cultivation of AOA on solid media

*‘Ca*. N. franklandus C13’ and *N. viennensis* EN76 were grown on solid medium using the Liquid - Solid method (LS-method). The LS-method consists of a solid-phase (Phytagel™) in which the cells are embedded and a liquid-phase in which the gel-embedded cells are submerged. The LS-method involves four main steps (i) cell harvesting (ii) gel preparation (iii) inoculation and (iv) maintenance. Experiments were performed in triplicate in acid-washed 100 ml Duran glass bottles. The method was repeated five times and has been validated independently in our laboratory.

Cells were harvested aseptically from a mid-late exponential culture onto a 0.2 µm pore size membrane filter (PES, Millipore) using a vacuum filtration manifold. Cells were resuspended in a sterile solution of FWM basal salts. A four-fold (1:10) serial dilution series of this cell suspension was performed.

Phytagel™ (0.6% (w/v)) was dissolved in FWM basal salts (containing phosphates) and sterilized by autoclaving at 121°C for 15 min (15 psi). All heat-labile medium supplements including streptomycin (50 µg mL^−1^) were added once the molten gel had cooled to approximately 42°C. Ammonia concentration was 5 mM and 3 mM for '*Ca*. N. franklandus C13' and *N. viennensis*EN76, respectively. A 20–25 ml aliquot of the molten Phytagel™ was transferred to each 100 ml Duran bottle and allowed to set.

The second Phytagel™ layer was prepared as above but inoculated with 50–80 µl of the diluted cell suspension (i.e. 10^−1^ (∼10^7^cells mL^−1^) or 10^−4^ (∼10^4^ cells mL ^−1^)) prior to solidifying. The Phytagel™: cell mixture was gently swirled to ensure even distribution of the cells. Once the inoculated Phytagel™ layer had solidified, it was overlayed with 20 - 25 ml of FWM medium containing streptomycin (50 µg mL^−1^). Note: A single Phytagel™ layer may also be used. The bottles were incubated in the dark at 37°C under static conditions.

Nitrite accumulation in the top liquid layer was measured to monitor growth of the cells in the gel. The bottles were also monitored for a change in colour of the pH indicator (phenol red) from pink to yellow indicating acidification of the medium. Once nitrite accumulation had ceased, the liquid layer was regularly replaced with the same volume of fresh medium.

### Nucleic acid extraction

Cellular biomass growing within the Phytagel™ was harvested aseptically using a sterile glass Pasteur pipette. Samples from three replicates were pooled (approximately 100 µl) and resuspended in 100 µl of FWM basal salts. DNA was extracted using a phenol:chloroform:isoamyl alcohol (25:24:1) protocol as described previously (Tourna *et al*. 2010). The DNA pellet was resuspended in 50 µl of TE buffer containing 100 mM Tris-HCl (pH 8) and 0.1 mM EDTA and stored at  -20°C.

### Polymerase Chain Reaction (PCR)

PCR products for the archaeal and AOA-specific 16S rRNA gene were generated using the primer sets A109F/1492R and 771F/957R, respectively (Table S2). The *‘Ca*. N. franklandus C13*’* specific urease gene (*ureC* subunit) was amplified using the primer set ureC1F/ureC1R (Table S2). PCR targeting the bacterial 16S rRNA gene was performed with primer set 27F/1492R (Table S2). PCR reactions were performed in 50 µl volumes containing: 1X Q5 polymerase reaction buffer, 0.2 mM dNTP mixture, 0.4 µM of each primer, 0.02 U/µl units of Q5 high-fidelity DNA polymerase (New England BioLabs, UK). All primers and PCR thermocycling parameters are listed in Table S2. All PCR generated sequences were analysed using BioEdit sequence alignment editor v.7.2.5 (Hall 1999) and the NCBI BLAST tool (Altschul *et al*. 1990).

### Fluorescence *in-situ* hybridisation (FISH) and transmission electron microscopy (TEM)

FISH analysis was performed according to the method previously described by Daims *et al*. (2005). A detailed technical description of sample preparation and visualisation for both FISH (Table S1) and TEM analysis may be found in the Supplementary section.

## Results

### Effects of different solidifying agents on AOA growth

**Figure 1.**
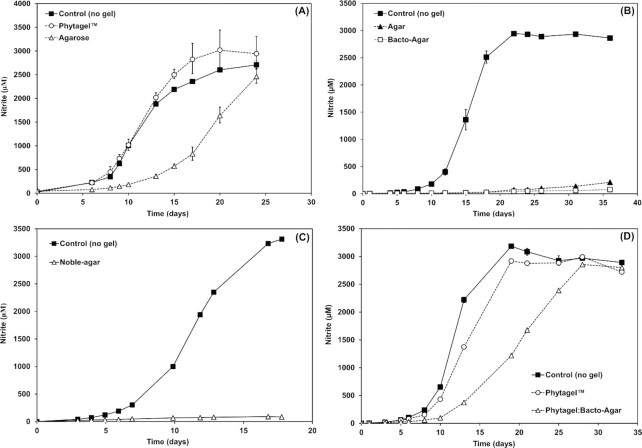
Effects of different solidifying agents on the growth of *‘Ca*. N. franklandus C13’: **(A)** Phytagel™ and agarose **(B)** agar and Bacto-agar **(C)** Noble-agar and **(D)** Phytagel™ and Bacto-agar (50:50) mixture. The control cultures were grown in the absence of a solidifying agent. Nitrite concentrations plotted represent the average of three replicate cultures. Error bars are standard errors of the means and may be smaller than the size of the symbol.

Three AOA strains were grown in the presence of different solidifying agents to determine their effect on growth. In the presence of Phytagel™, *‘Ca*. N. franklandus C13’ grew at comparable rates to the control cultures with growth rates of 0.47 d^−1^ and 0.40 d^−1^, respectively (Fig. 1A). In comparison, agarose resulted in a mild decrease in the growth rate of *‘Ca*. N. franklandus C13’ cultures (0.22 d^− 1^). These observations were in sharp contrast to agar, Bacto-agar and Noble-agar that all severely inhibited nitrite accumulation in *‘Ca*. N. franklandus C13’ cultures (Fig. 1B and C).

To further validate the toxic effects of Bacto-agar, a blended mixture (50:50) of Phytagel™: Bacto-agar was prepared. In this gel mixture, the growth rates of the *‘Ca*. N. franklandus C13*’* cultures declined to 0.34 d^−1^ compared to 0.45 d^-1^ in pure Phytagel™. This strongly suggested the presence of a toxic component in the Bacto-agar that negatively affects growth.

The effects of Phytagel™ and Bacto-agar on growth were further tested on *N. viennensis* EN76 and the acidophilic *‘Ca*. N. sinensis Nd2’ (Table 1, Fig. S1). The growth rates of *N. viennensis* EN76 in Phytagel™ (0.56 d^−1^) and the control (0.54 d^−1^) were comparable to the pattern observed in *‘Ca*. N. franklandus C13’. In contrast, the acidophilic *‘Ca*. N. sinensis’ Nd2 was virtually unable to grow in the presence of both solidifying agents. These data are summarised in Table 1.

**Table 1.** The effects of different gelling agents on the growth of AOA cultures.

Strain	Agar	Bacto-Agar	Agarose	Phytagel™	Noble-Agar
*‘Ca*. N. franklandus C13’	**-**	-	+	++	-
*N. viennensis* EN76	NT	-	**-**	**++**	NT
*‘Ca*. N. sinensis Nd2’	NT	-	NT	-	NT

NT: Not tested

(−): Poor/no growth

(+): Moderate growth

(++): No effect on growth

### Cultivation of AOA on solid medium

Phytagel™ was selected as a suitable solidifying agent for both *‘Ca*. N. franklandus C13’ and *N. viennensis* EN76 cultures as it did not negatively affect their growth. We proceeded to develop the LS-method to produce single colonies from both strains. The LS-method involved inoculating AOA cells into molten Phytagel™ and allowing this mixture to solidify. The solidified cell:Phytagel™ layer was subsequently overlayed with liquid medium. Cultures were monitored by measuring nitrite accumulation in the top liquid layer (Fig. S2).

*‘Ca*. N. franklandus C13’ and *N. viennensis* EN76 cells growing within the gel accumulated nitrite (Fig. S2). This agreed with the pre-screening experiments and confirmed that the cells within the Phytagel™ were indeed actively growing. In addition, a change in colour of the liquid medium from pink to yellow, indicated acidification, consistent with ammonia oxidation.

### Colony development

**Figure 2.**
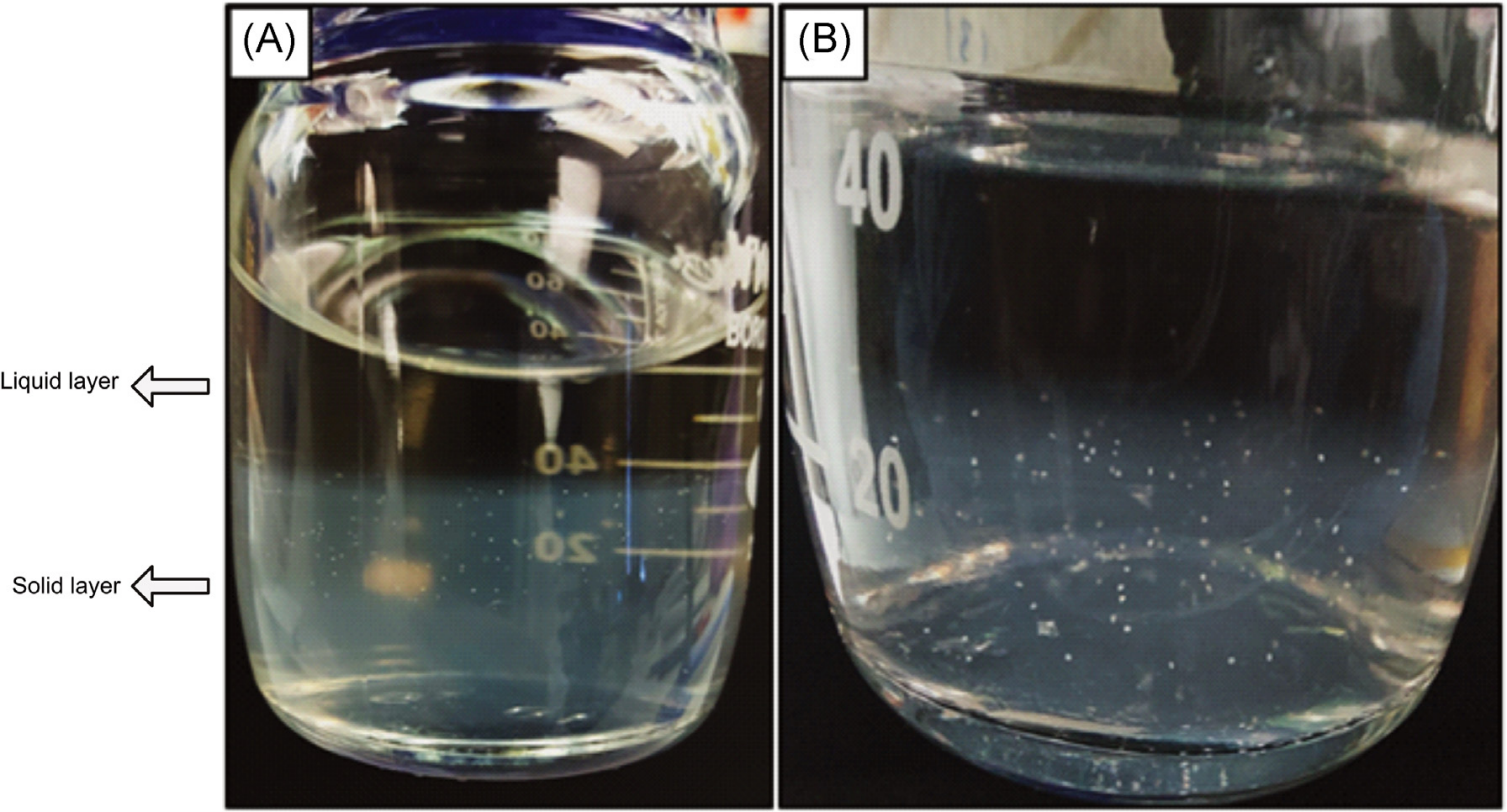
*‘Ca*. N.  franklandus C13’ colonies developing in Phytagel™. Colonies are grown and maintained in 100 ml Duran glass bottles, and measure <1 mm in diameter.

Colonies developed from *‘*Ca. N. franklandus C13’ and *N. viennensis* EN76 cultures once the liquid-phase had been replaced approximately twice (Fig. 2 and S3). The high-density inoculum resulted in a lawn of colonies that took approximately 4–5 weeks to appear. In comparison, colonies from the low-density inoculum took a longer time to appear at about 7–8 weeks. The colonies from both strains were white/off-white in appearance and gradually became more distinct with regular replacement of the medium.

### Plating efficiency

The plating efficiency for ‘*Ca*. N. franklandus C13’ was determined as a measure of the number of colonies observed to the number of cells plated (number of colonies counted/number of cells plated). The number of colonies ranged between 100 and 154. Based on the number of cells inoculated, a plating efficiency of >85% was determined, indicating that majority of the cells formed colonies. This supports the gel-toxicity tests which showed that Phytagel™ did not impact growth of *‘Ca*. N. franklandus C13*’*.

### Sequence analysis of colonies

PCR amplification of the 16S rRNA gene from the *‘Ca*. N. franklandus C13’ colonies, using both archaeal and AOA-specific primers, generated bands of the expected size i.e. ∼1,383 bp and ∼200 bp respectively (Fig. S4). A BLASTn analysis of these amplicons revealed a 100% and 99.5% nucleotide identity respectively (query cover:100%) to the 16S rRNA gene sequence in the published genome of *‘Ca*. N. franklandus C13’ with the accession number LR216287.1. The query sequences used ranged between 1247 bp (archaeal) and 190 bp (AOA-specific) in length. In addition, we amplified the urease alpha subunit gene (*ureC*) specific to *‘Ca*. N. franklandus C13’. A BLASTx analysis using a 119 bp sequence, revealed a 100% amino acid identity to the urease alpha subunit (UreC) from *‘Ca*. N. franklandus C13’ with the accession number WP_145988037.1.

### Fluorescence microscopy

**Figure 3.**
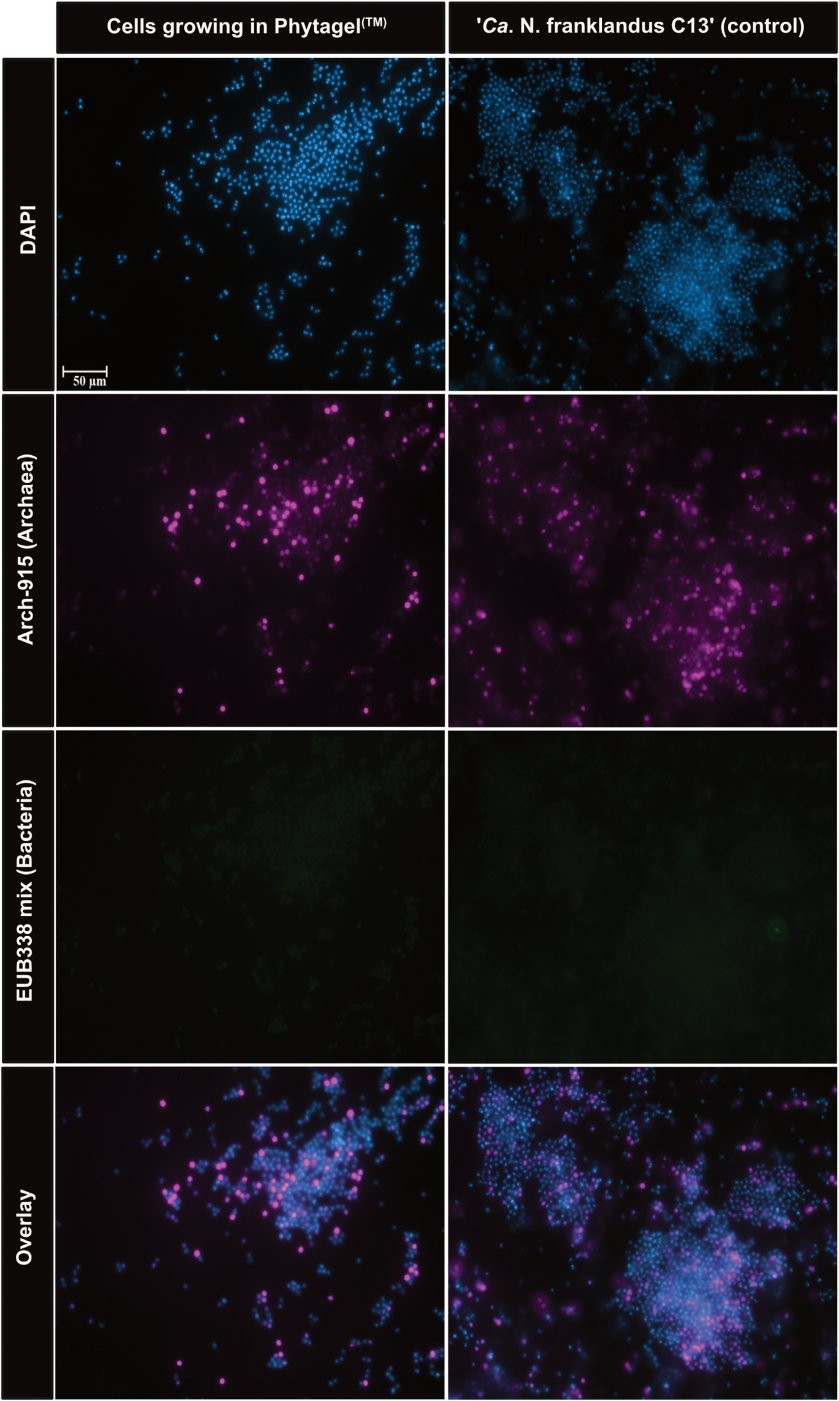
Fluorescent micrographs of cells growing with the Phytagel^TM^. Cells growing within Phytagel^TM^ (left column) and cells from a pure liquid culture of ‘*Ca*. N. franklandus C13’ as a control (right column). Cells from a pure culture of *Nitrosomonas europaea* ATCC 19178 were used as a control (not shown). Cells are stained with FISH probes for archaea (ARCH-915, violet), bacteria (EUB338 mix, green) and DAPI (blue). Images were viewed at 1000 X magnification.

Fluorescent *in situ* hybridisation (FISH) using archaeal probes was used to confirm the identity the colony-forming cells within the Phytagel™ (Fig. 3). The counterstain DAPI (blue), indiscriminately labels all cells within the sample and revealed irregular cocci, a characteristic cell morphology of *‘Ca*. N. franklandus C13’. A signal from the archaeal probes (violet) confirmed the presence of archaeal cells and overlapped with the DAPI signal. The lack of signal from the bacterial probes (green) suggests the absence of bacterial cells demonstrating a homogenous population of archaeal cells within the Phytagel™.

### Transmission electron microscopy (TEM)

**Figure 4.**
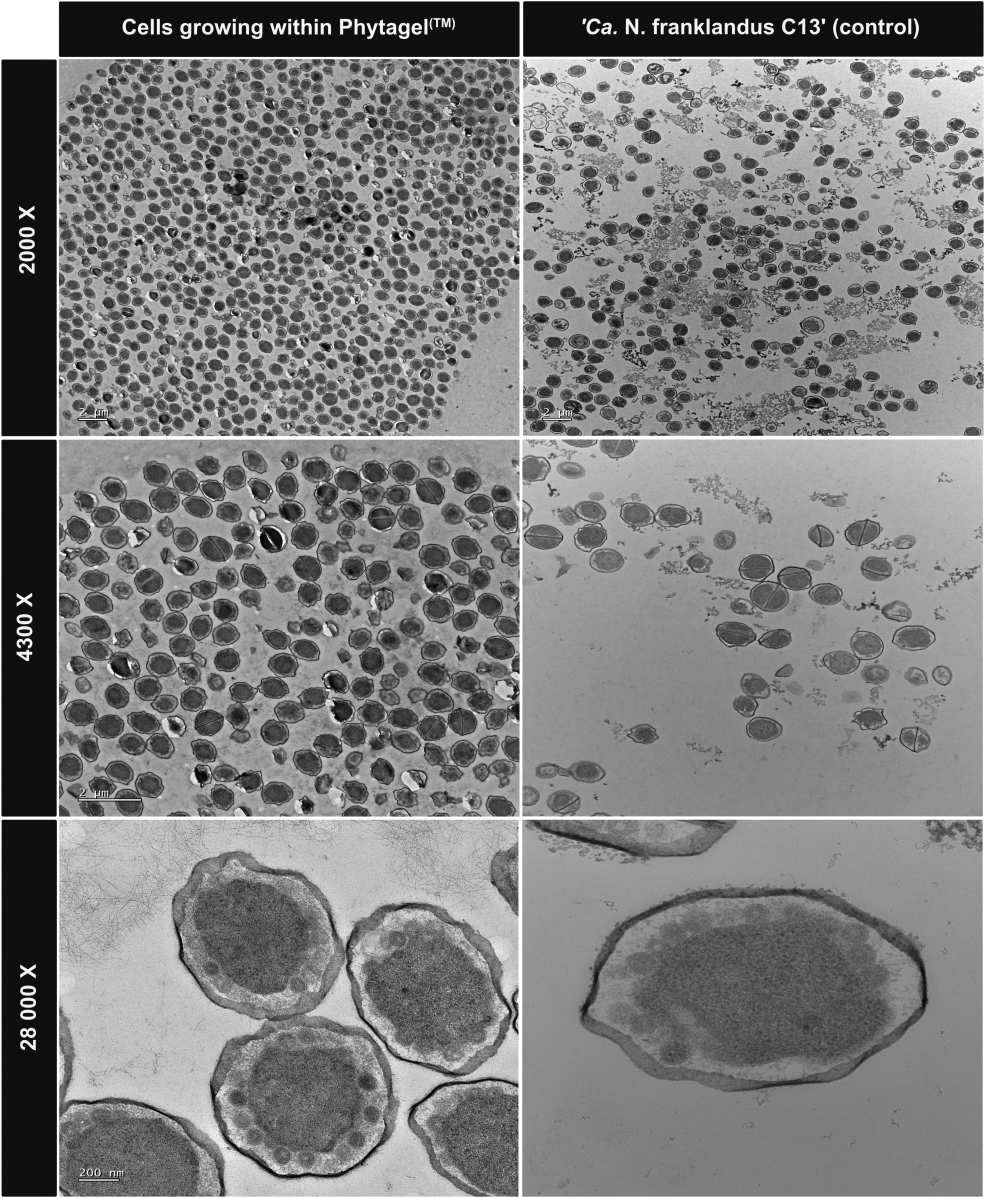
Transmission electron microscopy of cells growing within Phytagel^TM^ (left column) and cells from a pure liquid batch grown culture of ‘*Ca*. N. franklandus C13’ (right column).

Colonies growing within the Phytagel™ were sampled and visualised with high-resolution TEM. The cell morphology and internal structures were compared to cells from a pure liquid batch culture of *‘Ca*. N. franklandus C13’ as a control (Fig. 4). At low magnification (2000 X), a homogenous population of irregular cocci was observed which is typical of this AOA strain. At higher magnification (28,000 X), we were able to distinctly visualise the unusual vesicle-like structures characteristic of *‘Ca*. N. franklandus C13’, the function and identity of which is currently unknown. Nonetheless, these data indicate that the cells growing within Phytagel™ share both internal and external structural similarity to the reference sample.

### Subculturing from AOA colonies

**Figure 5.**
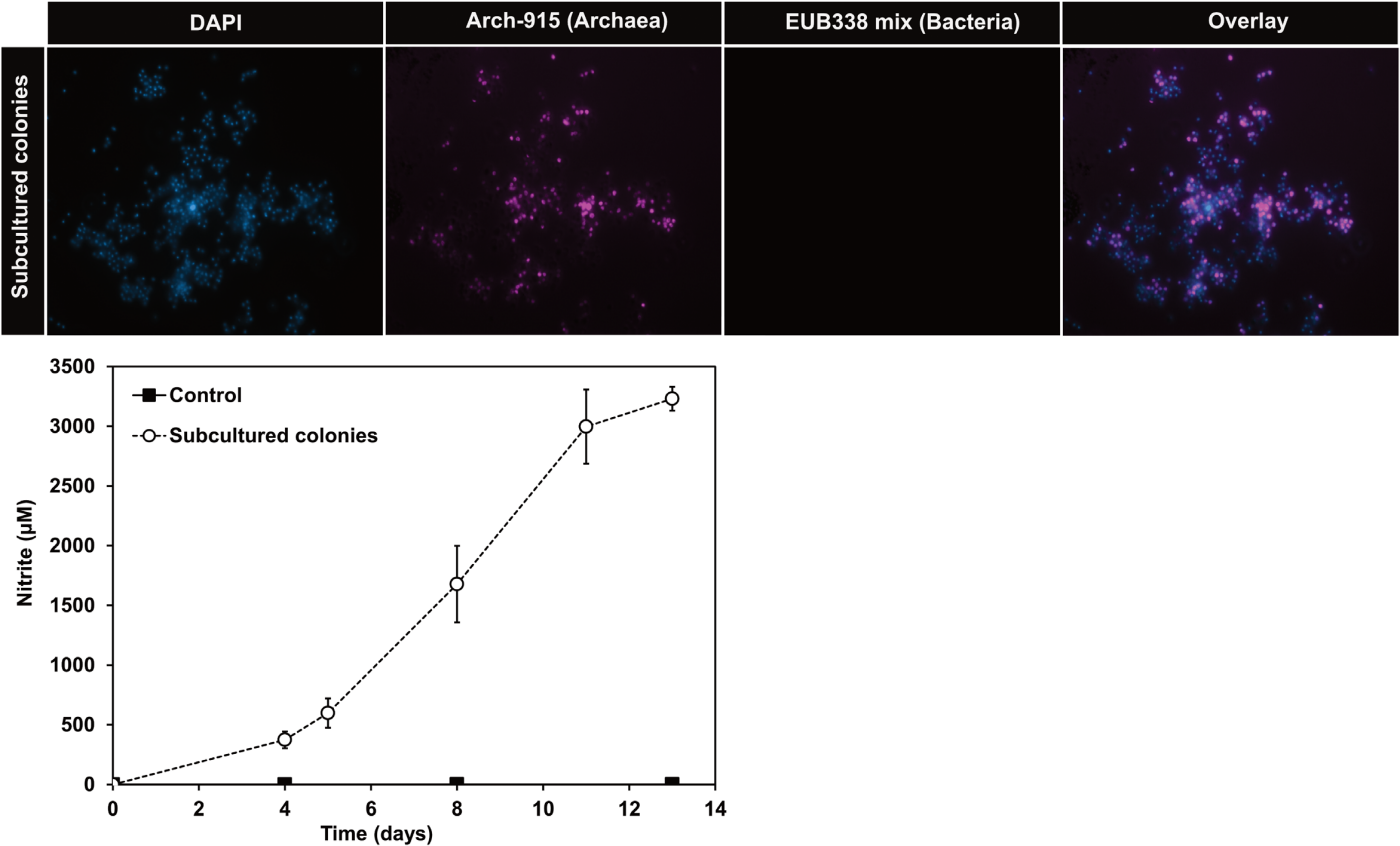
Fluorescent micrographs of cells grown from a colony of ‘*Ca*. N.  franklandus C13’. Cells were stained with FISH probes for archaea (Arch-915, violet), bacteria (EUB338 mix, green) and DAPI (blue). Images were viewed at 1000 X magnification. The graph shows nitrite accumulation from the subcultured colonies. Nitrite concentrations were measured in triplicate and error bars are standard errors of the means and may be smaller than the size of the symbol.

A small sample of the gel-embedded cells were scraped and transferred to fresh liquid medium and nitrite accumulation measured (Fig. 5). Nitrite accumulation in all three replicates indicated the presence of viable and actively growing ammonia-oxidising cells. Further analysis of the resulting biomass using archaeal FISH probes also demonstrated the presence of a homogeneous population of AOA cells. A signal from the bacterial probes was not detected.

## Discussion

Our findings highlight and emphasize the importance of selecting a suitable solidifying agent for the cultivation of AOA on solid medium. Although all AOA strains tested were inhibited by agar-based gels, the response to agarose and Phytagel™ varied between strains. Inhibition of microbial growth by solidifying agents such as agar has been well documented and is attributed to various factors including the production of hydrogen peroxide and the release of free sugars. This may be important as some AOA strains lack catalases, which normally detoxify hydrogen peroxide (Qin *et al*. 2014, Kim *et al*. 2016). Sand and Bennett (1966) also discuss numerous case studies on the variable effect that agar has on microbial growth based on the source of the agar as well as the brand. Other possible contributors to growth inhibition by agar is the presence of sulfated polysaccharides which exhibit a range of bioactive properties (Farias *et al*. 2000, Ngo and Kim 2013). Evidently, there is a large number of possible reasons for the inhibition of the AOA strains by agar-based gels that warrant further investigation, but are beyond the scope of the present study.

Phytagel™ is the trade name for gellan gum, an exopolysaccharide produced by *Sphingomonas elodea* (Vartak *et al*. 1995). Gellan gum is a tetrasaccharide composed of repeating units of D-glucose (2), L-rhamnose (1) and D-glucuronic acid (1) (Kang *et al*. 1982, Das *et al*. 2015). Both *‘Ca*. N. franklandus C13’ and *N. viennensis* EN76 tolerated Phytagel™ well, whilst *‘Ca*. N. sinensis Nd2’ did not. Reasons for inhibition of the acidophilic AOA strain are unclear but may be linked to the low pH and requires further testing. It is interesting to note that gellan gum was previously used to successfully isolate an ammonia-oxidising bacterium (AOB) from soil (Takahashi *et al*. 1992). Phytagel™ is an excellent alternative to agar as it exhibits desirable physical properties such as high optical clarity and gel strength (Jaeger *et al*. 2015). We emphasise that the growth of the AOA strains in this study on Phytagel™ was dependent on the batch and brand used (data not shown). The inability to reproduce previously published data upon changing to a new batch of Phytagel™ has also been reported. Differences in hardness between Phytagel™ batches, which may influence the reproducibility were observed (Jacques *et al*. 2020). Nonetheless, our data highlight the need to pre-screen solidifying agents prior to cultivation of AOA on solid medium.

The accumulation of toxic levels of nitrite, coupled with the slow growth rates, likely contributes to the difficulty in cultivating AOA on solid medium. To overcome this issue, we have developed the Liquid-Solid approach (LS). The principle behind the LS-method, which consists of a biphasic growth matrix, is that the nitrite excreted by the AOA cells within the gels diffuses into the overlaying liquid phase which is subsequently aspirated off and replaced with fresh medium. This overcomes or at the very least minimises the toxic accumulation of nitrite, thus facilitating the continuous growth of the cells.

In this study, we have demonstrated that the LS-approach facilitates the development of visible distinct single colonies from AOA cultures. Furthermore, a high plating efficiency (>85%) has been determined for *‘Ca*. N. franklandus C13’ which is highly desirable in the context of developing a genetic system. It is also possible to pick the single colonies produced from the low-density inoculum using a thin sterile glass Pasteur pipette. However, due to the three-dimensional growth of the colonies within the gel, picking single colonies is challenging as the pipette may easily contact nearby colonies.

To validate that the colonies indeed originate from AOA cells, we employed both molecular and microscopy-based methods. Cells growing within the gel were labelled with FISH probes targeting archaeal and bacterial 16S rRNA. A signal was detected for the archaeal-specific probes but not for the bacterial probes indicating the presence of only archaeal cells. The cells exhibited a cellular morphology (irregular cocci) identical to the cells from a pure culture of *‘Ca*. N. franklandus C13’. However, the use of the Cy3-labeled Arch-915 probe resulted in a weak fluorescent signal from the *‘Ca*. N. franklandus C13’ cells as observed in Fig. 3. This may result from low rRNA content and poor cell permeability (Wagner *et al*. 2003). Therefore, FISH was coupled with high-resolution TEM, PCR and sequence analysis which all indicate that the cells are *‘Ca*. N. franklandus C13’. A very faint band was amplified with the bacterial primers which is most likely transient DNA as no signal was detected from the bacterial FISH probes and no bacterial cells could be discerned from the high-resolution electron micrographs. In addition, no bacterial cells were detected by FISH from the colonies that were subcultured.

The LS-method has potential future applications for pure culture isolation of AOA which is currently restricted to time-consuming enrichment procedures. For example, if a dilute sample of a highly enriched AOA culture is grown using the LS-method and visible colonies are produced, then it may be possible to pick a desired colony expediting the purification process. This approach may also be useful for the purification of bacteria capable of the complete oxidation of ammonia to nitrate via nitrite also known as comammox. Currently a single pure culture of a comammox exists, which hinders their thorough physiological characterisation (Daims *et al*. 2015, Sakoula *et al*. 2020). The ability to produce single colonies from AOA is a major advance in facilitating the development of a genetic system for these archaea.

A main caveat with the LS-method is that it is seemingly not applicable to acidophilic AOA. Further work is necessary to better understand the inhibition of Phytagel™ and agar-based gels on *‘Ca*. N. sinensis Nd2’. Also, it is difficult to determine colony counts due to their three-dimensional arrangement. We also observed the presence of free-floating cells in the liquid phase presumably due to the detachment from the gel-matrix. Lastly, although visible colonies from the high-density inoculum took on average 5–6 weeks, colonies from the low-density inoculum took a longer time (about two months) to reach a size that can easily be picked.

## Conclusion

In conclusion, we have demonstrated that selection of a suitable solidifying agent is crucial for the successful cultivation of AOA on solid medium. Furthermore, we describe here a novel method for the cultivation of *‘Ca*. N. franklandus C13’ and *N. viennensis* EN76 on solid medium that results in visible and distinct colonies. The relative simplicity and no requirement for specialised equipment makes the LS-method accessible to any microbiology laboratory working with this group of microbes.

## Supplementary Material

fnac029_Supplemental_File
