# Supplementary material for: Cultivation of ammonia-oxidising archaea on solid medium
Source: FEMS Microbiol Lett. 2022 Mar 22;369(1):fnac029. doi: 10.1093/femsle/fnac029 (PMC9072212; doi:10.1093/femsle/fnac029)
Supplement: fnac029_Supplemental_File [file fnac029_supplemental_file.docx]

**Supplementary information**


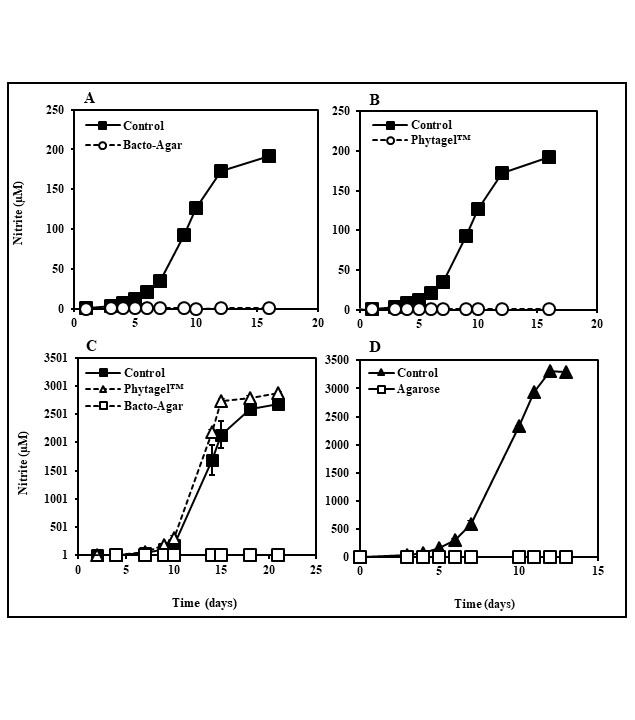


**Figure S1:** Effects of Phytagel™ and Bacto-agar on the growth of ‘*Ca.* N. sinensis Nd2’ (**A and B**) and *N. viennensis* EN76 (**C and D**). The control cultures were grown in the absence of a solidifying agent. Nitrite concentrations represent the average of three replicate cultures. Error bars are standard errors of the means and where they are not visible are smaller than the size of the symbol.

**Figure S2:** Evidence of actively growing AOA cells embedded in Phytagel™. Nitrite was measured in cultures containing the high-density inoculum. **(A)** Nitrite accumulation by ‘*Ca*. N. franklandus C13’ cells growing within Phytagel™. Nitrite concentrations are the averages of three replicate cultures. Error bars represent the standard error of the means and may be smaller than the symbol **(B)** Nitrite accumulation by *N. viennensis* EN76 cells embedded in Phytagel™. Nitrite concentrations for three replicate cultures are plotted individually. (C) Evidence of media acidification based on colour change of phenol red pH indicator. Control (left) and high-density inoculum of ‘*Ca.* N. franklandus C13’ (right).

**Figure S3:** Photographs of *N. viennensis* EN76 colonies growing within Phytagel™. (A) Colonies arising from a high-inoculum and (B) Colonies arising from a low-density inoculum. Fluorescent micrographs of *N. viennensis* EN76 cells growing within the Phytagel™. Cells are stained with FISH probes for archaea (Arch-915, red), bacteria (EUB338 mix, green) and DAPI (blue). Images were viewed at 1000 x magnification.

**Figure S4:** Amplification of **(A)** archaeal 16S rRNA gene **(B)** AOA-specific region of the 16S rRNA gene **(C)** ‘*Ca*. N. franklandus C13’-specific urease gene (*ureC* subunit) and **(D)** bacterial 16S rRNA from cells growing within Phytagel™. Lanes **(L)**: 1kb λ ladder, **(P)**: Positive control, **(N)**: Negative control (blank), **(E)**: Genomic DNA extracted from colony biomass and **(C)**: blank control cultures. The PCR amplicons were resolved on a 1% (w/v) agarose gel.

| **Table S1: Oligonucleotide probes used in this study** | | | |
| --- | --- | --- | --- |
| **Probe** | **Target** | **Sequence (5’ → 3’)** | **Reference** |
| Arch-915  (Cy3-labelled) | Archaea | GTGCTCCCCCGCCAA TTCCT | (Stahl & Amann, 1991) |
| EUB338 1  EUB338 2  EUB338 3  (Fluos-labelled) | Most bacteria  Planctomycetales  Verrucomicrobiales | GCTGCC TCC CGTAGGAGT  GCAGCCACCCGTAGGTGT  GCTGCCACCCGTAGGTGT | (Daims, *et al*., 1999)  (Amann, *et al*., 1990) |

| **Table S2: Primer pairs and PCR thermocycling parameters** | | | | |
| --- | --- | --- | --- | --- |
| **Primer** | **Sequence (5’ → 3’)** | **Target** | **Thermocycling parameters** | **Reference** |
|  |  |  |  |  |
| 771F  957R | ACGGTGAGGGATGAAAGCT  CGGCGTTGACTCCAATTG | AOA-specific 16S rRNA | 95°C for 5 mins, 30 cycles of 95°C for 30 sec, 55°C for 30 sec, 72°C for 30 sec and a final extension at 72°C for 10 min | (Ochsenreiter *et al*, 2003) |
|  |  |  |  |  |
| ureC1F  ureC1R | AAGGAGCTGGTGGAGGTCAC  AATGCGACGTCCTCTGCAAC | *‘Ca.* N. franklandus C13’-specific urease (*ureC*) | 98°C for 30 sec, 25 cycles of 98°C for 10 sec, 56°C for 30 sec, 72°C 20 sec and a final extension 72°C for 2 min | (This study) |
|  |  |  |  |  |
| 27F  1492R | AGAGTTTGATCCTGGCTCAG  GGTTACCTTGTTACGACTT | Bacterial 16S rRNA | 95°C for 3 min, 30 cycles of 95°C for 30 sec, 55°C for 45 sec, 72°C for 1:20 sec and a final extension at 72°C for 5 min | (Lane, 1991) |
|  |  |  |  |  |
| A109F  1492R | ACKGCTCAGTAACACGT  GGTTACCTTGTTACGACTT | Archaeal  16S rRNA | 95°C for 5 min, 30 cycles of 95°C for 1 min, 55°C for 1 min, 72°C for 1:30 min and a final extension at 72°C for 6 min | (Grosskopf *et al*, 1998) |

## Transmission electron microscopy (TEM)

Plugs containing Phytagel™ embedded cells were sampled with a wide-bore tip and placed in a sterile solution of FWM. The wide-bore tips were prepared by simply cutting off the tip of a 1ml pipette tip with a sterile blade. The gel plugs were cut into 1 mm^2^ cubes with a surgical blade and fixed overnight using a 2.5% (v/v) solution of glutaraldehyde in 0.05 M of sodium cacodylate (pH 7.3) at 4°C. A stationary liquid-batch culture of *‘Ca.* N. franklandus’ C13 was used as control. Prior to fixation, the cells were embedded in agarose using glutaraldehyde at a final concentration of 2.5% (v/v). The cells were pelleted by low-speed centrifugation and most of the supernatant discarded. The cell pellet was mixed with an equal volume of 2% (w/v) low-melting point agarose at 37°C and briefly plunged in ice. Low-melt point agarose containing cells was cut into cubes measuring 1 mm^2^. These cubes containing concentrated cells were fixed overnight in a solution of 2.5% (v/v) glutaraldehyde in 0.05 M sodium cacodylate (pH 7.3) at 4°C.

The fixed samples were embedded using a Leica EM TP embedding machine (Leica, Milton Keynes, UK). The fixative was washed out with three successive 15 min washes in 0.05 M sodium cacodylate and post-fixed in 1% (w/v) osmium tetroxide (OsO_4_) in 0.05 M sodium cacodylate at room temperature. The cells were washed thrice with distilled water. Ethanol dehydration was performed in an increasing ethanol series (30%, 50%, 70%, 95% and two times in 100%) for 1 hr each. Following dehydration, samples were gradually infiltrated with LR White resin (London Resin Company, Reading, Berkshire) by successive changes of resin:ethanol mixes at room temperature (1:1 for 1 hr, 2:1 for 1 hr, 3:1 for 1 hr, 100% resin for 1 hr then 100% resin for 16 hours and a fresh change again for a further 8 hours). The samples were transferred into gelatin capsules containing fresh LR White and incubated at 60°C for 16 hrs to polymerize.

Ultra-thin sections of approximately 80 nm were cut with a glass knife using a Leica UC7 ultramicrotome (Leica, Milton Keynes, UK). The sections were picked up on 200 mesh copper grids coated with Formvar and carbon film (EM Resolutions, Sheffield, UK). Sections were stained with 2% (w/v) uranyl acetate for 1 hour and 1% (w/v) lead citrate for 1 min and subsequently washed with distilled water and air dried. The grids were viewed in a FEI Talos 200C transmission electron microscope (FEI UK Ltd, Cambridge, UK) at 200 kV and imaged using a Gatan OneView 4K x 4K digital camera (Gatan, Cambridge, UK) to record DM4.

## Fluorescent *in-situ* hybridisation (FISH)

Cells growing within the Phytagel™ were harvested using the tip of a sterile glass Pasteur pipette. Samples from three replicates were pooled totalling approximately 100 µl of the Phytagel™:cell mixture to which an equal volume of sterile FWM solution was added. Gel-embedded cells were released by vortex mixing for 10 min. Phytagel™ pieces were removed by centrifugation at 2000 x *g* for 5 min. The supernatant was transferred to a fresh tube and centrifuged at 16 000 x *g* for 40 - 60 min to harvest the cells. The biomass was resuspended in 20 µl of 1X Phosphate buffer saline (PBS) buffer and fixed with 3 volumes of 4% (v/v; in 1X PBS) formaldehyde (ACROS organics, USA) at room temperature for 30 min. Fixed cells were harvested at 16 000 x *g* for 15 min and washed with 500 µl 1X PBS buffer. The washed and fixed cells were finally resuspended in 50 - 100 µl of a 50:50 mix of PBS:absolute ethanol and stored at -20°C.

Fixed cells (10 µl) were spotted onto Teflon coated slides (Thermo Scientific, USA) and oven dried at 46°C for 10 min. The samples were dehydrated in an increasing ethanol series of 50%‑80%‑100% for 3 min each. A 35% (v/v) formamide hybridization-buffer was prepared containing: 0.9 M NaCl, 20 mM Tris and 0.001% (v/v) SDS. To each well, 10 µl of the hybridization-buffer and fluorescent probes (Table S1) specifically targeting AOA and bacterial 16S rRNA at a concentration of 3 ng/µl and 5 ng/µl, respectively, was added. The fluorescent probes were hybridized overnight in an air-tight chamber at 46°C. The slides were rinsed in washing-buffer containing 70 mM NaCl, 20 mM Tris-HCl (pH 8) and 5 mM EDTA for 15 min at 48°C. Lastly, the slides were briefly plunged in ice-cold MilliQ water and dried at 46°C for 1 min. Cells were embedded in Vectashield mounting solution containing 4′,6-diamidino-2-phenylindole (DAPI) (Vector Laboratories Inc., Burlingame, CA, USA). Images were acquired with a Zeiss AXIO Scope.A1 microscope and analysed with the Zen (2011) v. 1.0.0.0 software (Carl Zeiss, Germany).

**Plating efficiency**

Cells were enumerated by microscopic cell counts with DAPI stain as previously described (Lehtovirta-Morley *et al*., 2014). Ten fields of view were visualised and counted at 1000 x magnification on a Zeiss AXIO Scope.A1 microscope (Carl Zeiss, Germany). Colonies were counted in three individual replicate bottles. Plating efficiency was calculated by dividing the number of colonies by number of cells inoculated.
